# Which Trauma Treatment Suits me? Identification of Patients' Treatment Preferences for Posttraumatic Stress Disorder (PTSD)

**DOI:** 10.3389/fpsyg.2021.694038

**Published:** 2021-08-12

**Authors:** Laura Schwartzkopff, Jana Gutermann, Regina Steil, Meike Müller-Engelmann

**Affiliations:** Department of Clinical Psychology and Psychotherapy, Institute of Psychology, Goethe University Frankfurt, Frankfurt, Germany

**Keywords:** PTSD, trauma, treatment preference, psychotherapy preferences for PTSD treatment, psychotherapy

## Abstract

Several psychotherapy treatments exist for posttraumatic stress disorder. This study examines the treatment preferences of treatment-seeking traumatized adults in Germany and investigates the reasons for their treatment choices. Preferences for prolonged exposure, cognitive behavioral therapy (CBT), eye movement desensitization and reprocessing (EMDR), psychodynamic psychotherapy and stabilization were assessed *via* an online survey. Reasons for preferences were analyzed by means of thematic coding by two independent rates. 104 traumatized adults completed the survey. Prolonged exposure and CBT were each preferred by nearly 30%, and EMDR and psychodynamic psychotherapy were preferred by nearly 20%. Stabilization was significantly less preferred than all other options, by only 4%. Significantly higher proportions of patients were disinclined to choose EMDR and stabilization. Patients who preferred psychodynamic psychotherapy were significantly older than those who preferred CBT. Reasons underlying preferences included the perceived treatment mechanisms and treatment efficacy. Traumatized patients vary in their treatment preferences. Preference assessments may help clinicians comprehensively address patients' individual needs and thus improve therapy outcomes.

## Introduction

People with posttraumatic stress disorder (PTSD) suffer from a variety of distressing symptoms, such as recurrent painful memories of the traumatic event, nightmares, severe physical tension, changes in mood and thoughts, and sleeping disorders (American Psychiatric Association, 2013). Moreover, PTSD is associated with high comorbidities and particularly high rates of comorbid major depression and anxiety disorders (Galatzer-Levy et al., 2013). The lifetime prevalence of this severe disorder ranges from 8 to 11% in the U.S. population (Kilpatrick et al., 2013), whereas a prevalence of 1.9% has been reported in Europe (Alonso et al., 2004). Studies investigating representative samples of the German general population have demonstrated lifetime prevalence rates between 1.4 and 2.9% (Hapke et al., 2006; Hauffa et al., 2011). Overall, the personal and social costs associated with PTSD are enormous, and PTSD causes the highest economic burden among all anxiety disorders (Marciniak et al., 2005). Psychotherapy has been demonstrated to be the gold standard in the treatment of PTSD and has been shown to be more effective than psychopharmacotherapy (Cusack et al., 2016). Among the diverse therapeutic approaches (Cusack et al., 2016), psychological treatments for PTSD can mainly be divided into two branches: trauma-focused interventions and non-trauma-focused interventions (Watkins et al., 2018). Whereas *trauma-focused interventions* aim to directly address the traumatic event [e.g., eye movement desensitization and reprocessing (EMDR; Shapiro, 2001), exposure therapy (Foa and Rothbaum, 1998), and cognitive behavioral therapy (CBT; Ehlers and Clark, 2000)], the goal of *non-trauma-focused methods* is to treat PTSD symptoms without directly addressing the traumatic event and associated memories (e.g., relaxation and stabilization) (Watkins et al., 2018). Stabilization, for example, aims to empower the traumatized patient to deal with strong emotions and the trauma-related burden in general (Reddemann and Piedfort-Marin, 2017). Trauma-adapted psychodynamic therapies are also part of the psychotherapeutic care of traumatized patients, but the evidence for their efficacy is insufficient (Paintain and Cassidy, 2018). Meta-analyses and guidelines have identified trauma-focused psychological interventions as the most effective treatments for PTSD (Cusack et al., 2016; American Psychological Association, 2017), with the strongest evidence of effectiveness especially for prolonged exposure, CBT and EMDR (Lewis et al., 2020). Despite the wide range of treatment options and the immense burden and distress associated with PTSD, a large proportion of PTSD patients do not respond to empirically supported treatments, with non-response rates sometimes as high as 50% (Schottenbauer et al., 2008; Sripada et al., 2019). If PTSD is treated too late or even remains untreated, the risk of chronification increases drastically (Zlotnick et al., 1999). Therefore, it is very important to better understand the factors that can lead to improved treatment compliance and response. In their everyday practice, clinicians are obliged to inform patients about the various treatment options and encouraged to address patients' treatment preferences to promote a shared decision making (Kwan et al., 2010). Previous research has demonstrated that considering patients' treatment preferences can improve treatment outcomes (Le et al., 2018; Swift et al., 2018; Delevry and Le, 2019; Windle et al., 2020). Even though there is a growing body of literature assessing the treatment preferences of PTSD patients, prior research is limited. So far, the main focus of previous research on the treatment preferences of PTSD patients has been to determine patients' preferences for pharmacological interventions vs. psychological interventions (mainly prolonged exposure). A review of 41 studies on preferences for trauma treatment found that, overall, participants preferred psychotherapy over medication (Simiola et al., 2015). Concerning different psychotherapy options, findings from this review identified exposure therapy to be the most frequently assessed treatment option (*n* = 16, 40%), followed by CBT (*n* = 12, 30%). Only a small proportion of studies assessed preferences for EMDR (*n* = 4, 10%) or psychodynamic psychotherapies (*n* = 4, 10%) (Simiola et al., 2015). In general, studies comparing psychotherapeutic approaches show that preferences for the different psychotherapy treatment options for PTSD varied across previous studies, but participants were most likely to choose exposure therapy and cognitive therapy over other approaches, such as psychodynamic therapy or EMDR (e.g., Tarrier et al., 2006; Becker et al., 2007, 2009; Etingen et al., 2020; Harik et al., 2020). In addition, studies have demonstrated that non-standard therapies (i.e., computerized treatment, coaching, family therapy, and alternative therapies) appear to be less preferred than established treatments, such as CBT or exposure therapy (Najavits, 2015; Schumm et al., 2015; Simiola et al., 2015). Demographic factors such as sex, age, level of education, previous treatment and psychopathology variables have been examined as possible influencing factors on treatment choice, but the findings are inconclusive. For example, Markowitz et al. (2016) compared the treatment preferences of 87 chronic PTSD patients for interpersonal psychotherapy (50%), prolonged exposure (26%) and relaxation therapy (26%). Patients with a preference for prolonged exposure reported more temporal distance from the primary trauma, a higher prevalence of sexual abuse and more depressive episodes in the past. In contrast, in a sample of 74 trauma-exposed women, Angelo et al. (2008) reported a higher number of years of education as the single factor to be associated with an increased likelihood of choosing prolonged exposure. Similarly, Chen et al. (2013) demonstrated that neither demographic factors nor treatment or trauma history were predictors of treatment preference. As demographic and psychopathological variables do not seem to provide a consistent explanation for the choice of treatment, a qualitative approach examining individuals' reported reasons for their treatment preferences seems to be of greater benefit and predictive value (e.g., Cochran et al., 2008; Chen et al., 2013). Previous studies mainly focused on reasons for the choice between medication (sertraline) and one psychotherapy (prolonged exposure) but did not examine preference reasons for different types of psychotherapy. Overall, the most commonly cited reasons for the preference for medication vs. psychotherapy included the perceived treatment mechanism, the expected efficacy of the treatment, and the potential side effects or health concerns (Angelo et al., 2008; Chen et al., 2013). Evidence indicated that the reasons for choosing prolonged exposure therapy were primarily based on the perceived treatment mechanism, whereas individuals choosing pharmacotherapy appeared to place greater emphasis on the perceived efficacy of the treatment (Angelo et al., 2008; Chen et al., 2013). To our knowledge, only one prior study covered the preference reasons for more than one PTSD treatment (i.e., cognitive processing therapy, prolonged exposure, EMDR, stress inoculation training, medication) (Etingen et al., 2020). The study concluded that the perceived effectiveness was the most frequently reported preference driver, followed by the treatment mechanism and the perceived personal fit (Etingen et al., 2020). However, previous studies were either limited by their use of non-clinical samples that were given a hypothetical trauma scenario (e.g., Zoellner et al., 2003; Tarrier et al., 2006; Becker et al., 2007) or, for those studies analyzing clinical samples (e.g., Angelo et al., 2008; Schumm et al., 2015; Markowitz et al., 2016; Miles and Thompson, 2016), by the lack of inclusion of more than two of the specifically highly recommended first-line psychotherapy options (i.e., EMDR, CBT, prolonged exposure; American Psychological Association, 2017; Lewis et al., 2020). Recent preference studies overcome those limitations by examining multiple recommended treatment options for PTSD treatment (Etingen et al., 2020; Harik et al., 2020). But in these studies, participants did not represent treatment seeking PTSD patients, meaning that their treatment choices were hypothetical. Furthermore, there is a lack of research exploring PTSD patients' preferences for stabilization and psychodynamic psychotherapy, that are often used in clinical practice, which is especially true for some European countries like Germany and France (Reddemann and Piedfort-Marin, 2017; Paintain and Cassidy, 2018). Moreover, prior research lacks the combination of comprehensive treatment preference data and qualitative data exploring underlying reasons for those preferences (Etingen et al., 2020).

To our knowledge, no studies to date have examined treatment preferences and reasons for these preferences in treatment seeking PTSD patients considering the most highly recommended treatment options (i.e., EMDR, CBT, prolonged exposure), as well as psychodynamic therapy and stabilization, which are quite popular in clinical practice. Additionally, as far as we know, there are no studies that combine comprehensive treatment preference data and qualitative data with respect to the reasons for those preferences.

The present study therefore aims to expand previous research by investigating (a) the treatment preferences of PTSD patients with respect to five evidence based (EMDR, CBT, exposure) or frequently used (psychodynamic therapy and stabilization) psychotherapeutic treatments for PTSD, (b) the influence of demographic and psychopathological factors on patients' treatment preferences, and (c) the reasons for treatment choice.

Following previous research (e.g., Tarrier et al., 2006; Becker et al., 2009; Simiola et al., 2015; Harik et al., 2020) we assumed that a higher proportion of PTSD patients would indicate a preference for CBT and exposure therapy compared to the other treatment options. As findings on the influences of demographic and clinical variables on treatment preference are heterogeneous, and the reasons for the choice of these treatment options have not been studied in treatment seeking PTSD patients yet, we adopted an exploratory approach to address research questions b and c.

## Methods

### Participants and Recruitment

Patients were recruited from a specialized PTSD outpatient center in Germany from April 2018 until March 2019. All patients who applied for treatment after having experienced a traumatic event were screened for this study. We included patients who were age ≥ 18, had an experience of a traumatic event according to criterion A for PTSD in the Diagnostic and Statistical Manual of Mental Disorders (DSM-5; American Psychiatric Association, 2013) and were seeking treatment due to the consequences of this traumatic event. All patients gave written informed consent. Patients received 15€ compensation for their participation. The exclusion criteria were acute schizophrenic disorders, acute suicidal tendencies and acute threat to others. Patients were also excluded if another mental disorder was the main reason for seeking treatment.

In total, 140 patients were screened for the study. According to our exclusion criteria, *n* = 11 participants were excluded (*n* = 1 due to having acute suicidal tendencies, *n* = 5 due to not seeking PTSD treatment, *n* = 4 due to not meeting criterion A, and *n* = 1 due to having schizophrenic disorder). The total sample consisted of 129 patients. The study received approval from the local ethics committee.

### Procedure

After the informed consent procedures, personal interviews were conducted. Interviews were performed during the initial consultation at the outpatient center and were carried out by licensed psychotherapists or psychotherapists in training. Following the personal interview, participants received an e-mail with a link and a personal code for the second part of the study—the online questionnaire. They were asked to fill out the questionnaire within 3 days after the interview. The online survey was carried out using Unipark (QuestBack GmbH). In the online survey, patients were presented with descriptions of 5 treatments in a random order and asked about their prior experience with each treatment. Afterwards, all treatment descriptions were presented together on the screen, and patients were asked to make a hypothetical choice regarding which treatments they would prefer and refuse (preferences did not play a role in the actual treatment they later received). Subsequently, patients were asked to list the reasons for their treatment choices. Patients were also asked to rate the severity of their PTSD symptoms [Posttraumatic Stress Disorder Checklist for DSM-5 (PCL-5)] and their degree of depression [Beck Depression Inventory-II (BDI-II)] in the online questionnaire.

### Materials and Measures

#### Interview

The interview comprised questions concerning sociodemographic variables, prior experiences with psychotherapy and treatment refusal as well as a short exploration of the traumatic event. Information about traumatization was assessed based on the Life Events Checklist (LEC-5) (Weathers et al., 2013).

#### Psychopathology Measures

##### PTSD

The *severity* of PTSD symptoms was assessed with the PCL-5 (Weathers et al., 2013). The PCL-5 is a 20-item self-report measure with items corresponding to the symptoms of PTSD listed in the DSM-5. The 20 items are rated on a 5-point Likert scale (0 = “not at all” to 4 = “extremely”), with a total symptom score of 80. A cut-off score ≥ 33 indicates a clinically relevant PTSD diagnosis. The PCL-5 has shown good reliability and validity (Blevins et al., 2015). In the present sample, Cronbach's alpha was α = 0.91. To assess exposure to a PTSD Criterion A traumatic event and to determine the index event, the LEC-5 (Weathers et al., 2013) was used. If several events were mentioned, the index event was determined by the following question: “Which is the most stressful event for you today?”

##### Depression

To assess depressive symptoms, the BDI-II (Beck et al., 1996) was used. The BDI-II is a 21-item questionnaire that assesses depressive symptoms over the past 2 weeks. Answers are given on a 4-point scale with at least four answers of increasing intensity to choose from. Possible total scores range from 0 to 63. According to the current cut-offs, 0–13 points indicate no or minimal depressive symptoms, 14-19 points indicate mild depressive symptoms, 20–28 points indicate moderate depressive symptoms, and 29–63 points indicate severe depressive symptoms (Beck et al., 1996). The BDI-II has shown good reliability and validity (Wang and Gorenstein, 2013). Cronbach's alpha was α = 0.93.

#### Treatment Options and Reasons for Treatment Choice

##### Treatment Options

Patients were presented with descriptions of five treatments for PTSD, all widely used in psychotherapeutic care (CBT, exposure therapy, EMDR, psychodynamic psychotherapy and stabilization). The treatment descriptions were developed by experienced clinicians according to previous studies (Tarrier et al., 2006; Becker et al., 2007) and were reviewed by experts in the respective field. The treatment descriptions contained information about each therapeutic model, the treatment duration, scientific evidence and known advantages and disadvantages of the specific treatment. Even though the descriptions were not all exactly the same length, efforts were made to keep the lengths balanced. The mean word count of the descriptions was 223.6 words (SD = 22.37; range = 190–250). The detailed therapy descriptions can be found in Supplementary Material.

All five treatment descriptions were listed side-by-side on the screen in random order. To assess treatment preferences, the following forced-choice question was asked: “If you had the choice between prolonged exposure, cognitive behavioral therapy, EMDR, psychodynamic psychotherapy and stabilization therapy to reduce the symptoms associated with the trauma (e.g., nightmares, disturbing thoughts, anxiety), which therapy would you choose?” The same procedure was used for treatment refusal: “Which of the described therapies would you most likely reject in order to reduce the symptoms associated with the trauma?”

##### Reasons for Treatment Choice

To understand the reasons that influenced the patients' treatment preferences, an open question was used to ask participants which reasons had influenced their choice. To systemize the responses, five response fields were provided, and respondents were asked to order their five most important reasons from “1” (most important) to “5” (least important).

#### Development of Categories and Coding

To categorize the reasons for treatment choice, we used an inductive-deductive approach (Mayring, 2000). Thus, to begin the coding process, we used categories suggested in the previous literature (Zoellner et al., 2003; Angelo et al., 2008; Cochran et al., 2008; Chen et al., 2013) as a preliminary list.

Then, two trained raters (the first and the last author, both clinical psychologists) reviewed the answers on reasons for treatment choice and modified and extended the preliminary categories throughout the process. The analysis of the reasons for treatment preference revealed the following seven categories:

##### Treatment Efficacy

This category included all reasons related to the perceived ability of the treatment to reduce symptoms and the perceived ability of the treatment to work (e.g., “I think the treatment can help me reduce my symptoms”). Many responses assigned to this category focused on the perceived long-term effects of the treatment in reducing symptoms and the scientific proof for the treatment (e.g., “I read many positive studies about the treatment”; “The effectiveness of the treatment is scientifically proven”).

##### Treatment Effectiveness

This category related to reasons associated with the perceived positive benefit that could be achieved through the treatment, such as positive conditions and life changes (e.g., “I think this treatment makes it possible to improve my self-image;” “I think this treatment changes my whole life situation”).

##### Treatment Mechanism

A third category covered the perceived way in which the treatment worked to achieve treatment effects (e.g., “Confronting and dealing with the trauma seems important to me”; “To find out the unconscious effect of the trauma on me”).

##### Side Effects

A fourth category reflected reasons that were related to the perceived side effects of the treatment (e.g., “This therapy does not seem to be too exhausting and distressing”; “Less risk of temporary worsening”).

##### General Conditions

This category reflected the general conditions of the respective treatment, such as the intensity or duration of treatment (e.g., “I think this treatment would take less time”; “Long treatment duration, so you don't have to stress yourself”).

##### Previous Experiences/Recommendations

This domain included all reasons that related to previous experiences with the same or other treatment approaches, as well as treatment recommendations by others (e.g., “I read in a book about this therapy”; “Advice from my partner”).

##### Other

The last category included all reasons that could not be clearly categorized under the six domains mentioned above (e.g., “It is my gut feeling”; “Because it made the most sense to me”).

To obtain an overall impression of the reasons mentioned by a participant, all reasons written in the open-ended question were coded. In addition, the two raters also assigned the most important reason listed by each participant to one of the categories. Interrater reliability was calculated for the answers of 25% of the participants. The interrater reliability of the two independent coders was good across categories (*k* = 0.79) and for the primary reason for the treatment preference (*k*= 0.83).

### Statistical Analyses

Descriptive statistics were used to describe the sample characteristics and to evaluate the percentages of preferred or rejected treatments. To investigate whether the frequency of the preference of a treatment differed among treatments, we conducted a chi-square goodness-of-fit test and examined the 95% confidence intervals (CIs) for the percentages. If there is no overlap between 95% CIs, it can be assumed that there is a statistically significant difference between the means at an alpha level of 0.05. For simplicity, we treated overlapping CIs as non-significant differences, which is a common procedure (Schenker and Gentleman, 2001; Cumming and Finch, 2005; Tan and Tan, 2010).

We used cross-tabulation to examine the relationship of treatment preference to other categorical or nominal variables (e.g., sex, type of trauma, previous treatment experience), and to evaluate differences in reasons for preferences by type of treatment and clinical variables. The statistical significance of a crosstab was tested with chi-square tests of independence, or, in case of small cell sizes <5, with Fisher's exact test. One-way analyses of variance (ANOVAs) were conducted for continuous outcome variables (e.g., age, PTSD severity, depression severity), with treatment preference as the independent categorical variable. If the ANOVA results were significant, we followed up with Tukey *post hoc* tests. We calculated the respective effect sizes: *phi* for chi-square tests and eta-squared (η*2*) for ANOVAs. The data analysis was carried out using the statistics software SPSS 20 (IBM Corp, 2011). For the analyses, an alpha of 0.05 was used to determine statistical significance.

## Results

### Characteristics of the Study Sample

After the initial interviews, *n* = 25 (19.4%) participants dropped out of the study because they did not complete the online survey. Only participants with a complete data set were included. In total, we included 104 participants (78% female) in the study with an average age of 37.28 (*SD* = 12.15; range: 18–70) years. With respect to educational level, 32 (31%) had received 18 years of education, 23 (22%) had received 13 years of education and 49 (47%) had received 10 years or less of education. All participants described an event meeting the DSM-5 trauma criteria. The reported index events included sexual assault (35%) and physical assault (29.1%). For 86.5% of the sample, the index traumatic event was interpersonal violence, and 66.3% reported experiencing repetitive trauma. In 54.8% of the patients, the index traumatic event occurred during childhood. According to the PCL-5 scores, 74% of the participants met the criteria for a clinically relevant PTSD symptoms (*M* = 44.84; *SD* = 16.60). The mean BDI-II score in the entire sample was 26.35 (SD = 13.54). Most patients (70.2%) scored in the moderately or severely depressed range. With reference to previous treatment, 78.8% of the patients reported having previously sought treatment (outpatient and/or inpatient), but only 25% reported prior experience with PTSD-specific treatment. A total of 41.3% of the patients were taking psychotropic drugs at the time of the survey.

### Treatment Preference

The results of the treatment preferences and disclinations are shown in Table 1.

**Table 1:** Patient attitudes toward treatment: preferences and disinclinations.

	***n***	**%**	**95% CI**
**Treatment preference**			
Exposure therapy	34	32.7	[24; 42]
CBT	28	26.9	[18; 35]
Psychodynamic psychotherapy	19	18.3	[11; 26]
EMDR	19	18.3	[11; 26]
Stabilization	4	3.8	[0; 8]
**Disinclination**			
CBT	5	4.8	[1; 9]
Exposure therapy	8	7.7	[3; 13]
Psychodynamic psychotherapy	16	15.4	[8; 22]
EMDR	37	35.6	[26; 45]
Stabilization	38	36.5	[27; 46]

*CBT, cognitive behavioral therapy; EMDR, eye movement desensitization and reprocessing; CI, confidence interval. Based on N = 104 participants*.

Differences with respect to treatment preference were significant, χ^2^(4) = 24.75, *p* < 0.0001. Based on a comparison of the corresponding 95% CIs, the preference for stabilization (*n* = 4) was lower than the preferences for all the other treatment options (i.e., exposure therapy (*n* = 34), CBT (*n* = 28), psychodynamic psychotherapy (*n* = 19), EMDR (*n* = 19)). Differences for disinclination of a treatment were significant, χ^2^(4) = 47.83, *p* < 0.0001. Analysis of the 95% CIs revealed higher disinclinations for stabilization (*n* = 38) and EMDR (*n* = 37) compared to either CBT (*n* = 5), exposure therapy (*n* = 8) or psychodynamic psychotherapy (*n* = 16).

#### Factors Influencing Treatment Preference

##### Sociodemographic Variables

The results of the one-way ANOVA showed a statistically significant difference in the age of participants who preferred the different treatment options (*Welch's F* (4,19.17) = 3.32, *p* < 0.05, η^2^ = 0.09). Tukey's *post hoc* analysis revealed a significant age difference (*p* = 0.028) between participants who preferred psychodynamic psychotherapy (*M* = 43.32 years, *SD*= 8.53) and those who preferred CBT (*M* = 32.82 years, *SD* = 10.90). Sex [χ^2^(4) = 5.525, *p* =0.238], type of trauma (interpersonal vs. non-interpersonal) [χ^2^(4) = 7.690, *p* = 0.104], prior treatment experience [χ^2^(4) = 5.616, *p* = 0.230] and educational level [χ^2^(24) = 16.253, *p* = 0.879] showed no significant relations to treatment preferences or disinclinations for any treatment.

##### Clinical Variables

There were no statistically significant differences in severity of depression [*F*_(4, 99)_= 1.341, *p* = 0.260] or PTSD symptoms [*F*_(4, 99)_ = 0.520, *p* = 0.721] among patients with the different treatment preferences.

### Analysis of Reasons for Treatment Choice

#### Reasons for Treatment Choice

Almost half of the patients (41%, *n* = 43) cited the perceived treatment mechanism as the primary reason for their preferences. As an example, patients' reasons often reflected a desire to confront and deal with the trauma. Twenty-six percent (*n* = 27) cited treatment efficacy, reflecting the degree to which participants believed that the treatment is efficacious. Reasons under this domain also included ideas about the perceived long-term effects and scientific proof for the treatment. Another 16% (*n* = 17) cited other reasons as the primary reason. Treatment effectiveness was reported by 9% (*n* = 9), and previous experience was cited by 4% (*n* = 4) as the primary reason, whereas 2% cited side effects (*n* = 2) and 2% cited general conditions (*n* = 2) as the primary reason underlying their choices. A similar pattern emerged when all the given reasons were assigned to the categories: 67% of the participants mentioned the treatment mechanism, 53% mentioned the treatment efficacy, 22% mentioned other reasons, 21% mentioned treatment effectiveness, 17% mentioned general conditions, 15% mentioned side effects, and 10% mentioned previous experience as their reasons for their treatment choices.

#### Depression

When examining only respondents who scored in the moderately or severely depressed range on the BDI-II (*n* = 73, 70.2%), a similar pattern emerged for primary reasons. For individuals with BDI-II scores in the moderately or severe depressed range, 40% cited the treatment mechanism, 27% treatment efficacy, 12% cited other reasons, 12% treatment effectiveness, 4% mentioned previous experience, whereas 3% cited side effects and 1% cited general conditions as the primary reason underlying their choices. When comparing the occurrence of each of the specific reasons, there were no differences between individuals with depression scores in the moderately or severe range and individuals with depression scores in no or mild range, *p* = 0.189, Fisher's exact test.

#### PTSD

For individuals that met the criteria for a clinically relevant PTSD according to the PCL-5 (*n* = 77, 74%), 40% cited the treatment mechanism, 26% treatment efficacy, 14 % cited other reasons, 12% treatment effectiveness, 3% mentioned previous experience, 3% cited side effects and 3% cited general conditions as the primary reason underlying their choices. When comparing the occurrence of the specific reasons, there were no differences between individuals with clinically relevant PTSD and traumatized respondents without clinically relevant PTSD according to the PCL-5 (*n* = 27), *p* = 0.369, Fisher's exact test.

#### Reasons Cited for Each Treatment

When comparing the occurrence of specific reasons, the cited reasons for preference differed by type of treatment, *p* = 0.041, Fisher's exact test. The treatment-specific reasons are shown in Table 2.

**Table 2:** Proportions of reasons cited for each treatment.

	**Reasons for treatment preference**						
**Treatment preference**	**Efficacy** **(%)**	**Effectiveness(%)**	**Mechanism** **(%)**	**Side effects(%)**	**General conditions** **(%)**	**Previous experiences(%)**	**Other** **(%)**
Exposure(*n* = 34)	29	3	53	0	0	3	12
CBT(*n* = 28)	29	7	29	3	3	4	25
Psychodynamic(*n* = 19)	16	16	63	0	5	0	0
EMDR(*n* = 19)	26	10	16	5	0	11	32
Stabilization(*n* = 4)	25	25	50	0	0	0	0

*CBT, cognitive behavioral therapy; EMDR, eye movement desensitization and reprocessing. Based on N = 104 participants*.

A closer examination of the reasons associated with a treatment preference for prolonged exposure therapy suggests that the perceived treatment mechanism (53%; e.g., “Confronting the trauma seems important to me”) as well as the perceived treatment efficacy (29%; e.g., “This treatment is scientifically sound”) were important factors underlying treatment choice. Patients who chose CBT as their preferred treatment also cited especially reasons related to the perceived treatment mechanism (29%, e.g., “Correct thought patterns to speed up the healing process”) as well as reasons related to its perceived efficacy (29%, e.g., “Has long-lasting positive effects”). The respective treatment mechanism was also the most frequent cited reason for the choice of psychodynamic psychotherapy (63%; e.g., “To find out the unconscious effect of the trauma on me”). The most frequently mentioned reasons underlying a preference for EMDR were other reasons (32%, e.g., “New method I have not heard of before”) as well as reasons concerning treatment efficacy (26%). The perceived treatment mechanism was the most frequently cited reason for preferring stabilization (50%, e.g., “stabilizing”), although stabilization was chosen by only four participants.

## Discussion

This study aimed to investigate the preferences of patients suffering from PTSD symptoms for different psychotherapeutic treatments and to further analyze the influence of demographic and psychopathological factors on patients' treatment preferences and the reasons for their treatment choices. The combination of treatment preference data and qualitative data is the most unique feature of the present study. The five treatment options were prolonged exposure, EMDR, CBT, psychodynamic psychotherapy, and stabilization. Prolonged exposure and CBT were each preferred by nearly 30% of the sample, and EMDR and psychodynamic psychotherapy were preferred by nearly 20%. The proportion who preferred stabilization was lower, at only 4%. Stabilization was significantly less preferred than all other options. Significantly higher proportions of patients were disinclined to choose EMDR and stabilization than all other treatment options. Although stabilizing interventions are commonly applied in the German health care system (Neuner, 2008) and PTSD patients often receive long stabilization phases as standalone treatment, especially in inpatient settings (Lampe et al., 2008; Rosner et al., 2010), our findings indicate that a high rate of patient reject stabilization. Thus, there seems to be a gap between patient preferences and the reality in psychotherapeutic care. The patient preferences observed in this study correspond to findings that indicate only low to medium effects of stabilization alone on PTSD symptoms (Cloitre et al., 2002); together, these findings indicate that stabilization should be embedded in a phase-based approach instead of being used as a standalone intervention (Cloitre et al., 2011; Reddemann and Piedfort-Marin, 2017).

The higher disinclination rate for EMDR is consistent with previous research (Tarrier et al., 2006; Becker et al., 2007, 2009; Harik et al., 2020). Thus, Tarrier et al. (2006) examined preferences for 14 different PTSD treatment options, including cognitive therapy, cognitive therapy with exposure, psychodynamic psychotherapy and EMDR, in 330 undergraduate students. Participants rated cognitive therapy as the first-choice treatment and cognitive therapy with exposure as the second highest, whereas psychodynamic psychotherapy was ranked eight, and EMDR received the lowest rank. In summary, findings regarding EMDR point at a discrepancy between the high clinical recommendation and effectiveness of EMDR on the one hand (American Psychological Association, 2017) and the rather low preference of EMDR by PTSD patients on the other hand. In contrast to Tarrier et al. (2006), we could not find significant differences between the preference rates for exposure, CBT, EMDR and psychodynamic therapy. One reason could be the small sample size, which might have prevented differences from reaching statistical significance. Our findings are however in line with the conclusions of Tarrier et al. (2006) and Becker et al. (2007), who suggested, based on non-clinical samples, that there are overall equally strong preferences for both prolonged exposure and CBT but that preferences might differ on an individual level. Furthermore, in a larger study with 301 non-treatment seeking traumatized individuals, Harik et al. (2020) demonstrated that despite the relative preferences [e.g., cognitive processing therapy (43.6%), prolonged exposure (11.8%), EMDR (2.8%)], most participants were willing to consider each of the PTSD treatments. Since previous preference research with PTSD patients has mostly focused on comparisons between exposure therapy and medication (Angelo et al., 2008; Chen et al., 2013; Simiola et al., 2015), the results of the present study extend previous findings and indicate that trauma-focused therapy (e.g., exposure-based therapies) is broadly accepted by patients. Moreover, our findings indicate that treatments that have been shown to be effective in PTSD research (Cusack et al., 2016) are also desired by patients. On the other hand, our findings concerning psychodynamic therapy can also be interpreted in the light of previous work, pointing to an acceptance of non-exposure-based, more relationship focused psychotherapies (Markowitz et al., 2016).

We did not find individual differences, such as demographic and clinical factors, to be associated with treatment preference, except for age: people who preferred psychodynamic psychotherapy were significantly older than patients who preferred CBT. To date, previous treatment preference research has yielded inconsistent results concerning demographic and clinical variables as predictors of treatment preference; in some samples, demographic, and clinical variables, such as sex (Liddon et al., 2018) and educational level (Angelo et al., 2008), were found to be predictors of treatment preference, while in others, they were not (Chen et al., 2013; Markowitz et al., 2016). Recently, Harik et al. (2020) found that older patients more often preferred medication compared to younger participants. This contrasts with previous research suggesting that prolonged exposure therapy is well tolerated among elderly people (Thorp et al., 2019). To our knowledge, age was not reported to have predictive value in any other study, so future research might be necessary before drawing further conclusions.

Our results demonstrate that the perceived treatment mechanism, for example the desire to confront and deal with the trauma, played the most important role in influencing patients' treatment choice (41%). Treatment efficacy, reflecting the degree to which participants believed that the treatment is efficacious as well as ideas about the perceived long-term effects and scientific proof for the treatment, also emerged as a common reason underlying treatment preference (26%). This aligns with previous studies demonstrating the perceived mechanism and treatment effectiveness to be the most cited reason for treatment choice (Angelo et al., 2008; Cochran et al., 2008; Chen et al., 2013; Etingen et al., 2020). Our results indicate that frequencies of cited reasons differed by type of treatment but were not related to severity of PTSD or depressive Symptoms. Reasons associated with a treatment preference for prolonged exposure therapy suggests that the perceived treatment mechanism (53%; e.g., “Confronting the trauma seems important to me”) seems to be an important factor for preference. This finding is interesting since studies on clinicians' barriers to the implementation of prolonged exposure suggest that therapists are often afraid to overwhelm patients with the method (Becker et al., 2004). Our result particularly emphasizes the willingness of patients to deal with their trauma and is consistent with the current treatment preference literature (e.g., Zoellner et al., 2003; Jaeger et al., 2010). This result might reassure clinicians that trauma exposure and trauma-focused cognitive therapy is not only accepted but even strongly preferred by most patients. On the other hand, other PTSD sufferers preferred psychodynamic psychotherapy especially for its respective treatment mechanism (e.g., “To find out the unconscious effect of the trauma on me”). Among respondents with a preference for EMDR, its perceived efficacy was a frequently cited reason underlying the treatment choice. This finding is in line with previous research, indicating that especially reasons reflecting the perceived ability of the treatment to work seem to be important drivers underlying this preference (Etingen et al., 2020). Our findings support research approaches that stress that a “one size fits all” approach to therapy does not adequately consider individual patient needs and thus might diminish therapeutic success (Cloitre, 2015; Norcross and Wampold, 2018). To conclude, our study expands the existing literature on the treatment preferences of PTSD patients and highlights the need for patient-centered care, which includes informing patients about different treatment options. Although the patients who were surveyed sought treatment at a CBT-focused outpatient clinic, the analysis of their treatment preferences revealed that other treatment options were also considered preferable. This finding may indicate that treatment-seeking patients are often rather uninformed about different treatment options and, when provided with comprehensive information, might change their treatment preferences. For a better match between patient needs and evidence-based practice, it might be beneficial to inform patients about their general treatment options. Forward-looking, our results underscore the importance of the recommendations by Norcross and Wampold (2018), who suggested tailoring psychotherapy to the patient and his or her wishes, not only to the disorder, which is especially viable in PTSD treatment, as different treatment options have been proven to be effective (Watts et al., 2013). However, incorporating patients' treatment preferences into clinical practice will raise new challenges for clinicians that also need be considered in the future (Légaré and Thompson-Leduc, 2014).

### Limitations and Future Directions

Some limitations of the present study should be considered in the interpretation of the findings. Although we developed treatment descriptions according to the existing literature and with the help of external experts in the respective fields, the method of measuring treatment preference might have impacted the findings through the information presented in the texts as well as the word count of the descriptions. Concerning the word count, Tarrier et al. (2006) reported that the word count of treatment descriptions was not significantly associated with treatment preference, so it seems unlikely that the word count had a main impact in this study. It could further be argued that some treatments had an advantage over others because of their empirical evidence, which we however provided when available according to evidence-based-practice. Moreover, the format in which the treatment information was presented might have impacted patients′ treatment preferences. Nevertheless, the study by Harik et al. (2020) suggests that the format can influence patients' treatment acceptability but might not impact patients' first-choice selection. Apart from this, our study does not capture the option of not preferring any of the mentioned treatments or the case that a responded rated multiple treatment options as equally preferable or rejectable. Additionally, the self-selection of the assessed trauma patients might have influenced the results, as patients applied for standard PTSD treatment in an outpatient treatment center with a focus on CBT. Also, there may be a potential bias of sample selection and dropout, as 78% of participants who completed the study were female. Yet, according to a higher prevalence of PTSD in women than in men in the general population (Gavranidou and Rosner, 2003), the increased number of women in our sample can be seen as a reflection of the clinical reality. Furthermore, although we tried to develop the categories of the cited reasons for treatment preference based on the literature, the choice of our categories might have influenced the results. There is a possibility that the selected categories did not cover the material sufficiently or that the classification could have been performed differently. We tried to counter this issue by having two independent raters who demonstrated good interrater reliability. Further, we did not evaluate the actual treatment of participants and thus cannot draw conclusions about the impact of treatment preferences on treatment outcome. In the future, it would be important to further assess the relationship between patients' treatment preferences, the actual treatment they subsequently receive and the link of treatment success to preferences. Future research might also benefit from larger and more diverse patient samples from different inpatient and outpatient settings. Besides, it might also be of interest to assess the reasons for disinclination toward a specific treatment and to further provide a “no preference” option. Future research might also include the further examination of demographic and psychopathological factors influencing treatment preference. Since there are several evidence-based treatment options for PTSD (Lewis et al., 2020), it might be of further interest to investigate treatment preferences for PTSD by including additional trauma-focused treatments, such as Narrative Exposure Therapy (Schauer et al., 2011), or other non-trauma focused treatment options suggested as potentially useful, such as Interpersonal Psychotherapy (Weissman et al., 2000).

### Conclusion

As our study demonstrates that PTSD patients vary in terms of their treatment preferences, information and preference assessments may improve care for PTSD patients by allowing the provision of the optimal treatment for the individual, which might increase compliance and reduce dropout rates.

## Data Availability Statement

The raw data supporting the conclusions of this article will be made available by the authors, without undue reservation.

## Ethics Statement

The studies involving human participants were reviewed and approved by Local Ethics Committee, Department 05 Goethe-Universität Frankfurt on the Main. The patients/participants provided their written informed consent to participate in this study.

## Author Contributions

MM-E, JG, LS, and RS conceptualized the study. MM-E and LS collected, processed, and rated the data. LS performed the statistical analysis and prepared the manuscript. RS and MM-E supervised the research. All authors critically revised the manuscript revision and approved the final version.

## Conflict of Interest

The authors declare that the research was conducted in the absence of any commercial or financial relationships that could be construed as a potential conflict of interest.

## Publisher's Note

All claims expressed in this article are solely those of the authors and do not necessarily represent those of their affiliated organizations, or those of the publisher, the editors and the reviewers. Any product that may be evaluated in this article, or claim that may be made by its manufacturer, is not guaranteed or endorsed by the publisher.
