# Supplementary material for: Which Trauma Treatment Suits me? Identification of Patients' Treatment Preferences for Posttraumatic Stress Disorder (PTSD)
Source: Front Psychol. 2021 Aug 12;12:694038. doi: 10.3389/fpsyg.2021.694038 (PMC8387597; doi:10.3389/fpsyg.2021.694038)
Supplement: Supplementary file 1 [file Table_1.DOCX]

Supplementary Material

**1 Description of treatments**

**a) Exposure therapy**

This treatment is based on the assumption that posttraumatic stress disorder is essentially maintained by avoiding trauma-related thoughts, feelings and memories. During this treatment, the symptoms should be reduced by directly addressing (confronting) the memories of the traumatic experience. First, you work out with your therapist why this procedure is useful for reducing your symptoms. After you are sufficiently stabilized to become involved with this procedure, confrontation of traumatic memories takes place in several stages.
This includes a detailed report about the traumatic event as well as exercises in which you imagine the traumatic experience in your mind's eye. You have control over the course of the exercise at all times and are supported to feel safe in the here and now. After you achieve a reduced symptom burden, the next step is to go back to everyday situations that you are currently avoiding because they are associated with stressful memories.

Advantages: The effectiveness of this treatment has been proven in scientific studies. Overall, this treatment is very effective and has lasting positive effects. You will have the experience of being able to deal with the memories and being able to process the traumatic experience.

Disadvantages: The treatment can be very stressful because it requires dealing with memories and situations that you have previously avoided. Before a significant reduction in stress occurs, a temporary increase in burden may occur.

**b) Eye movement desensitization (EMDR)**

This treatment was named after right-left eye movements, which, like any back-and-forth movement, can have a calming and integrating effect. This effect can also be achieved by (right-left) alternating touches, e.g., of the hands, or listening to bilateral music (with sounds alternating between the right and left ear). Your therapist asks you, for example, to follow the movements of his finger with your eyes while thinking of the worst moment of traumatization. A stop signal is agreed upon, with which the exercise can be interrupted at any time. In a typical EMDR session, the therapist initially asks you to think about the worst moment you remember, a stressful thought about yourself, as well as a corresponding positive thought (e.g., "Today, I can defend myself"). After attention has been directed to feelings and body sensations, space is created for new inner images, thoughts, feelings and body sensations that may emerge spontaneously. The former experience can thus be experienced and placed in a new context. Each session ends with a deepening of the positive thoughts, e.g., "Today, I can defend myself" and the deepening of the positive body sensation.

Advantages: The effectiveness of this treatment has been proven in scientific studies. This treatment is generally very effective and has lasting positive effects for most people. This treatment does not require dealing with all details of the traumatic experience.

Disadvantages: Whether special eye movements or even mutual stimulation are necessary for the therapeutic effect is a controversial issue. The treatment can lead to slight headaches or nausea.

**c) Cognitive behavioral therapy (CBT)**

In this treatment, you will analyze, with the help of the therapist, stressful thoughts related to the trauma. People who have been traumatized often suffer from distorted distressing views, e.g., "I am to blame for the rape/attack", "I am not safe", and "My life is destroyed forever". These thoughts lead to feelings of guilt, shame, fear, etc. In the treatment, you will learn to recognize and challenge these beliefs and to find more balanced, appropriate beliefs again. Initially, you will receive substantial support from the therapist, but over the course of the treatment, you will gradually become an expert on your own problems. This procedure usually leads to a reduction in stressful feelings and thus of the symptoms of posttraumatic stress disorder.

Benefits: The effectiveness of this treatment has been proven in scientific studies. This treatment is overall very effective and has lasting positive effects. Although the traumatic experience is discussed in the treatment, the aim is not a direct and repeated confrontation with details of the trauma. Through the treatment, you will learn tools that will enable you to become your own therapist in the long term.

Disadvantages: It takes considerable effort to recognize and change distorted beliefs. This requires regular independent written homework between sessions. Often, challenged beliefs are understood from a rational point of view, but it takes a long time until the new thoughts feel right. Cognitive procedures are related to a certain amount of stress since traumatic experiences must be discussed to understand and change distorted thoughts.

**d) Stabilization**

Often, this treatment does not directly deal with the traumatic experience but rather aims to stabilize your psychological state. The aim of this is to make the burden bearable. This is achieved, for example, by working out techniques with your therapist to deal with strong tension, stress and burdensome feelings. In concrete terms, this may involve learning calming actions that help you distract yourself. It is also important to learn to recognize tension at an early stage and to deal with it actively. Furthermore, fantasy journeys or relaxation exercises can be used. For example, one exercise is the "safe place" exercise, in which you imagine a place where you feel safe and secure and to which you can return in your imagination when you are under heavy strain. Another exercise is the "thought stop", which is used to control recurring unpleasant thoughts.

Benefits: Pure stabilizing therapy involves very little stress, as there is no direct confrontation with traumatic experiences. This can make it easier to become involved in the treatment.

Disadvantages: In the treatment, the traumatic experience is not dealt with directly. According to studies, treatments that exclusively consist of stabilization have a low effectiveness and do not allow for long-term healing.

**e) Psychodynamic therapy**

The focus of psychodynamic trauma therapy is to identify and treat the unconscious effects of trauma on the patient. For example, it is determined how the trauma has changed the patient's personal values and what meanings are attributed to the experiences. It is assumed that the trauma is such an overwhelming experience that it could not be processed in the moment. In therapy, the conditions for processing are regained. Together with the patient, the therapist tries to understand to what extent current situations trigger trauma-related stress reactions, even though the traumatic event belongs to the past. The therapy will not focus mainly on symptoms but will primarily be concerned with establishing insight, which involves recognizing a connection between the burden of today and traumatic experiences. Here, the therapeutic relationship is of utmost importance, which means that feelings and behaviors that emerge within this relationship reflect problems in the patient’s daily life that can be addressed within the therapeutic relationship. Therefore, the therapist treats the patient with respect and support. Usually, such therapies take a long time.

Advantages: Psychodynamic therapies are widespread and do not require a detailed focus on traumatic memories.

Disadvantages: The therapy is long lasting. Currently, there is a lack of studies demonstrating the effectiveness of this treatment.

.
